# Extracellular Matrices as Bioactive Materials for In Situ Tissue Regeneration

**DOI:** 10.3390/pharmaceutics15122771

**Published:** 2023-12-13

**Authors:** Peng Zhao, Fengbo Yang, Xiaoli Jia, Yuqin Xiao, Chao Hua, Malcolm Xing, Guozhong Lyu

**Affiliations:** 1 Burn & Trauma Treatment Center, Affiliated Hospital of Jiangnan University, Wuxi 214122, China; 2 Engineering Research Center of the Ministry of Education for Wound Repair Technology, Jiangnan University, Affiliated Hospital of Jiangnan University, Wuxi 214000, China; 7222808024@stu.jiangnan.edu.cn (F.Y.); 6212809043@stu.jiangnan.edu.cn (Y.X.); 3 Department of Mechanical Engineering, University of Manitoba, Winnipeg, MB R3T 2N2, Canada

**Keywords:** nature-derived extracellular matrix, nano-structure, tissue engineering, acellular dermal matrix, urinary bladder matrix, small-intestinal submucosa

## Abstract

Bioactive materials based on a nature-derived extracellular matrix (NECM) represent a category of biomedical devices with versatile therapeutic applications in the realms of tissue repair and engineering. With advancements in decellularization technique, the inherent bioactive molecules and the innate nano-structural and mechanical properties are preserved in three-dimensional scaffolds mainly composed of collagens. Techniques such as electrospinning, three-dimensional printing, and the intricate fabrication of hydrogels are developed to mimic the physical structures, biosignalling and mechanical cues of ECM. Until now, there has been no approach that can fully account for the multifaceted properties and diverse applications of NECM. In this review, we introduce the main proteins composing NECMs and explicate the importance of them when used as therapeutic devices in tissue repair. Nano-structural features of NECM and their applications regarding tissue repair are summarized. The origins, degradability, and mechanical property of and immune responses to NECM are also introduced. Furthermore, we review their applications, and clinical features thereof, in the repair of acute and chronic wounds, abdominal hernia, breast deformity, etc. Some typical marketed devices based on NECM, their indications, and clinical relevance are summarized.

## 1. Introduction

Nature-derived extracellular matrix (NECM) refers to biological materials derived from tissues and organs of animals via decellularization. It is a complex mixture of proteins and other molecules, such as hyaluronic acid, forming a 3-dimensional (3D) network with nano-structures. In its innate form, ECM plays critical roles in regulating cell behaviors, such as adhesion, migration, differentiation, and proliferation [1,2]. In the decellularized form of native ECM, NECMs have been used as grafts to substitute damaged tissues or promote in situ tissue regeneration [3]. Nano-structural features, degradability, and the immune responses of NECMs are important factors influencing their performance as cellular scaffolds. This review is aimed at providing an overview of NECMs used as bioactive materials for in situ tissue regeneration. Therefore, the main molecules constituting NECMs and their contribution to the structural and biological properties of NECMs are briefly introduced. Nano-structures of NECMs, which in this article refer to scaffolds’ nanoscale morphology and nanopatterned interfaces, and their applications in tissue repair are discussed. Furthermore, the origins, degradability, and mechanical properties of and immune responses to NECMs are summarized. Based on this information, clinical applications of NECM grafts and evidence regarding their performance are introduced.

The intricacy of NECMs is partly embodied by their components. As of 2022, 2051 human matrisome proteoforms characterised by mass spectrometry have been included in MatrisomDB 2.0, a database integrating proteomic data on ECM composition [4]. Among them, structural proteins and proteoglycans concomitantly form the ECM “backbone”. The main structural ECM proteins are collagens, fibronectins, laminins, and elastins, which establish the three-dimensional nano-scaffolding space of ECMs. Proteoglycans fill the most interstitial part of this space. In addition to providing force-resistance and buffering, these molecules serve as a dynamic matrix in which cells reside and implement their functions [5] and, by inherently binding growth factors [6], provides an enzymatically accessible repository for these factors.

### 1.1. Collagens

**Figure 1:**
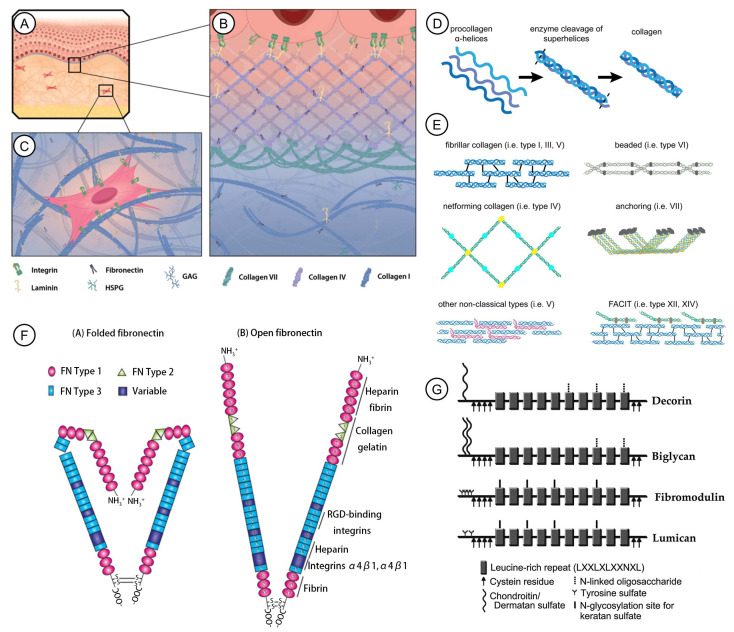
Mammalian skin nano- to sub-nano-structures as exemplary cell-ECM models and some of the dominant components of ECM. (**A**). Structure of mammalian skin. (**B**). Collagen type IV is the main structural components of the skin basal lamina. It sets a meshwork, together with fibronectin and laminin. (**C**). The dermis is built by collagen I and fibronectin, which form the three-dimensional fibrillary structure for cells to adhere to and migrate through. (**D**). Collagen is produced by the assembly of three α-helical procollagens and enzyme cleavage. (**E**). Different types of collagen molecules and their diverse structures. (**A**–**E**): Reprinted with permission from Ref. [7] under the terms of the Creative Commons Attribution License 2021, Pfisterer et al. (**F**). The open and folded confirmation of fibronectin. Reprinted with permission from Ref. [14] 2020, Drain et al. (**G**). Molecular structure of SLRP family members. Reprinted with permission from Ref. [16] 2014, Ni et al.

Collagens are a family of structural proteins used by cells for tissue integrity and other functions. The basic function of collagens is to provide mechanical support for cells, tissues, vessels, etc., and multidirectional resistance to forces (Figure 1A) [7]. They provide frames for the anchoring of multiple proteins such as laminins, cell surface receptors, and glycans, exerting versatile roles in regulating cell behaviors and maintaining tissue homeostasis (Figure 1B,C) [7,8]. Each collagen molecule contains three α-chains, each of which consists of repeating peptide triplets of glycine-X-Y and which together form a triple-stranded helix (Figure 1D) [7,9]. X and Y are in the form of proline and hydroxyproline, mostly, but can be any amino acid. The triple helical regions, designated COL domains, are flanked by non-Glycine-X-Y regions [10]. Through intra- and intermolecular crosslinks, collagen trimers further assemble into a staggered pattern with 67 nm D-band periodicity, generating collagen fibrils [11]. Oftentimes, the fibrils are made up of two or more collagen species. For instance, type V collagen is demonstrated to initiate the assembly of type I collagen molecules into fibril and regulate the diameter of the resulting fibril [12]. In addition to fibril-forming, other specific behaviors such as tethering to cell membranes and forming networks of the ECM are performed by other members of the collagen family. Collagen molecules are categorized, depending on their supramolecular architectures, into categories including fibrillar, fibril-associated containing interrupted triple helices (FACIT), network-forming, anchoring fibril, beaded collagens, and others with specific functions (Figure 1E).

### 1.2. Fibronectin

Fibronectin is a ubiquitous component of the ECM. It has a self-assembly domain at its N-terminal that enables the assembly of dimers into a three-dimensional matrix, which is an important condition for the deposition of other ECM networks [13]. The dimers are soluble when adopting a folded confirmation, which opens upon binding to other molecules (Figure 1F) [14]. Numerous other ECM components, including heparin sulfate, collagen, and fibrin, as well as certain membrane receptors in responsive cells, interact with fibronectin. Fibronectin can act as a mechano-regulator. Its cellular traction force-dependent unfolding reveals cryptic integrin-binding sites, giving rise to pleiotropic cellular alterations [15]. Fibronectin is involved in embryonic development, wound healing, and tumor metastasis.

### 1.3. Elastin

Elastin is the major ECM protein that provides elasticity and resilience to tissues. Elastic fibers are composed of heterogeneous complexes of microfibrils and tropoelastin monomers [17]. When subjected to cyclic loading, elastic fibers function as another key structural protein of the ECM, allowing soft tissues to revert to their initial form. It is worth noting that the tight connection between collagen fibrils and elastin limits elastin strain [18]. Elastic fibers may also facilitate cell signalling by acting as a reservoir for growth factors [19].

### 1.4. Laminins

Laminins are a family of ECM glycoproteins found in all animals. They are ubiquitous molecules in basement membranes and can also be found in other tissues [20]. Each laminin molecule consists of three polypeptide chains, namely α, β, and γ chains, which assemble into a heterotrimer. Laminins have high-affinity binding peptide domains to growth factors [20]. They are also capable of binding to integrins [21]. They have been shown to be critical for regulating cell adhesion, migration, and differentiation and for tuning matrix-mediated signalling, which is activated by the binding of integrin to the ECM [20]. They are implicated in a variety of biological processes including homeostasis, angiogenesis, wound healing, neural development, and tissue survival.

### 1.5. Other Molecules

Small leucine-rich proteoglycans (SLRPs) are a unique family of proteoglycans found in the ECM, including biglycan, decorin, and fibromodulin (Figure 1G) [16]. They mostly have both core proteins and glycosaminoglycan (GAG) chains, which are capable of binding cytokines and regulating ECM fibril assembly, spacing, and organization [22]. The inhibited expression of SLRPs leads to the abnormal formation of collagen fibril, as well as the delayed binding of cells to the ECM [23]. Moreover, SLRPs are able to sequester growth factors secreted in the ECM, such as basic fibroblast growth factor and bone morphogenetic proteins, with their GAG chains acting as co-receptors of these growth factors.

Perlecan is a heparan sulfate proteoglycan (HSPG) found to be rich in basement membranes [24]. Perlecan acts as a regulator of multiple biological processes, including angiogenesis, inflammation, and bone formation, and is thought to aid in vascular homeostasis [25]. It may also be involved in mechano-sensory communication between the cell and ECM [26]. Agrin is another significant HSPG in the ECM, originally found in neuromuscular junctions and later in numerous other locations. Motor neuron axons release neural agrin, which is able to stabilize neuromuscular junctions [27]. Non-neural splice variants of agrin are found in the kidney, lung, central nervous system, etc., and are suggested to link basement membranes and their underlying cells by virtue of the strong binding of agrin to laminin and a transmembrane protein linked to the cytoskeleton, α-dystroglycan [28]. Agrin may also be implicated in normal wound healing [29]. The bioactivity of perlecan and agrin is highly correlated with a type of GAG, heparan sulfate, which has binding sites with high affinity to cytokines, growth factors, enzymes, and other ECM components.

Hyaluronic acid is a large linear GAG containing repeating disaccharide units. Despite its structural simplicity, it performs crucial functions in cell signalling through binding to various receptors, including CD44 [30]. It has abundant negative charge and high molecular weight, rendering it attractive to large amounts of water and directly impacting the mechanical properties of tissues. It binds with chondroitin sulfate proteoglycans, concomitantly maintaining the integrity of specific tissues [14].

Lipids are present in the ECM of the stratum corneum and extracellular vesicles. Highly nonpolar lipids constitute an expanded ECM in which corneocytes are embedded, and this lipid matrix is responsible for the permeability barrier of the skin [31]. Lipids are the main molecular components of extracellular vesicles, and their composition and function in these vesicles is to be further investigated [32]. During osteoblast differentiation, proteolipids are required for the formation of extracellular vesicles, which promote bone mineralization [33].

## 2. Nano-Structures of ECM and Their Applications in Tissue Repair

One of the key features of native ECM is its nano-structures, which play important roles in cell adhesion, cell and tissue homeostasis, and the transmission of mechanical information. Changes in ECM nano-structures are implicated in tissue aging and disease, wound healing, and fibrosis [34,35,36]. They form 3D nanopatterned networks with intricate fibers that provide nanoscale binding sites, which are necessary for cell attachment [37]. Cells residing in the ECM are sensitive to changes in their surroundings, of which the nano-structures and topography have been shown to dictate cellular morphology, cell migration, and interactions with other cells [38,39]. By virtue of the nano-structures’ large surface area to volume ratio, the ECM shows numerous binding sites for cells as compared to scaffolds without nano-structures, enhancing cell/matrix interactions [40]. In addition, the individual size of transmembrane proteins that mediate cell attachment, such as integrins, is on the order of tens of nanometers, and cells exert piconewton-scale forces to their surroundings [41]. The stiffness and tensional states of cell surroundings are recognized through the bidirectional transmission of mechanical signals at nanoscale [42]. In native ECM, nano-structures are harnessed to mediate this transmission.

The most abundant nano-structures in human bodies are collagen nanofibers, which are the main ECM constituents of various tissue and organs, such as skin, tendons, cartilage, bone, and blood vessels. While maintaining the structural integrity of the tissues and organs, collagen nanofibers provide nanopatterned binding sites for cell binding and deliver tissue-specific mechanical signals to maintain cell homeostasis [35]. The length of type I collagen molecules is up to ~300 nm in diameter, and that of type III collagen molecules is shorter, ~100 nm in diameter. Both type I and III collagen molecules form microfibrils, which further assemble into fibrils. These fibrils are characterized by nanoscale periodic intervals, at the length scale of which cells may interact with collagen molecules [37]. Another molecule that forms nano-structures is fibronectin, which forms fibrils of ~2 nm in width. Studies on fibronectin fibrils have focused on the mechanisms of the force-triggered unfolding, which expose cryptic binding sites [43]. This mechanism is also of much relevance to NECMs used in tissue repair, because fibronectin is a main constituent of NECM. The mechanism of action of ECM nano-structures on cells is further complicated by flexible fibril sizes altered by the heterotypic assembling of various structural proteins and the nanopatterned distribution of various ligands in ECM [44]. Moreover, ECM topography created by all the nano-structures together is another significant aspect that regulates cell function [38].

Well-designed scaffolds have been developed to mimic the nano-structures of ECM. Methods employed in the fabrication of nanofibrous structures include electrospinning, thermally induced phase separation, and self-assembly [45]. Based on the nanofibrous structures, predesigned macropores, nanospheres loaded with drugs, proteins, and genes, and nanoscale inorganic materials have been incorporated to endow scaffolds with an improved ability to better mimic the properties of ECM [45]. Other strategies that have been developed to improve the performance of nanofibrous scaffolds include surface modification, the anisotropic aligning of nanofibers, and the incorporation of controlled drug delivery systems [46].

It is difficult to control the variable of nano-structural features of the NECM, due to its structural and compositional flexibility. Therefore, most evidence regarding the effects of nano-structures on cellular function comes from studies on synthetic nanofibrous scaffolds. As compared to solid-walled scaffolds, nano-structured scaffolds based on both NECMs and synthetic nanofibers enhance cellular functions, including cell attachment, proliferation, and differentiation. Nanofibrous poly(L-lactic acid) scaffolds absorbed 2.6–3.9 times more attachment proteins, including fibronectin and vitronectin, thus providing more binding sites for osteoblastic cells as compared to solid-walled scaffolds [47]. More human dermal microvascular endothelial cells adhered to hydrated small intestinal submucosa (SIS) than to type I collagen, type IV collagen, laminin, and fibronectin-coated plastic surfaces [48]. Moreover, nanofibrous scaffolds increased the expression of integrins with neonatal mouse osteoblasts as compared to solid-walled scaffolds [49]. MC3T3-E1 pre-osteoblasts, chondrocytes, and bone marrow-derived mesenchymal stem cells were reported to show enhanced proliferative ability on nanofibrous scaffolds [50,51,52]. Nanofibrous scaffolds have been demonstrated to enhance the differentiation and tissue-reconstruction function of several cell types such as chondrocytes [51], osteoblasts [47], and neural progenitors [53].

## 3. Importance of NECM

Although autograft transplantation is the clinical “gold standard” treatment for tissue defects, it requires an additional surgery that leads to co-morbidity and fibrosis. Moreover, under certain circumstances, autograft transplantation is inappropriate due to the shortage of healthy transplantable tissue and the inability to restore delicate structures of injured tissues in specialized locations, such as periorbital and mid-face regions [29].

The aim of tissue engineering is to repair tissue defects that cannot heal spontaneously and reconstruct new tissues to replace the lost or damaged tissue/organs. To achieve this aim, it is necessary to utilize matrices that provide biosignalling cues and mechanical support for cells to develop, organize, and behave as they are in their native ECM tissue both in vivo and in vitro [54]. Therefore, an ideal matrix for tissue engineering mimics the native environment of the ECM, providing biological and mechanical cues to allow cell attachment, migration, and differentiation [55].

Typically, matrices for tissue engineering are classified into two categories: artificial matrices and biological decellularized ECMs. Artificial matrices feature in the controllability of all their structural characteristics, including the porosity and the dimension [56]. However, the preservation of natural microenvironment features in decellularized ECMs is a notable advantageous property for their use in regenerative medicine, as compared with artificial matrices [57]. Importantly, the native ECM should be regarded as a dynamic structure built of various components such as ECM structural proteins, multiple growth factors, glycosaminoglycans, hormones, and other signalling molecules.

Many endeavors have been made to mimic the native ECM at nano-level by synthetic methods, one example of which is electrospinning [58]. The electrospinning approach is able to fabricate nano- to micro-fibers, which form interwoven networks, resembling the ECM fibrillar components [59]. Other methods holding remarkable promise include bioinspired hydrogels and 3D printing. Hydrogels enable the efficient encapsulation of cells, increasing cell seeding efficiency [60]. Their properties can be tuned to meet the accommodated needs for defects of differential tissues or organs. Three-dimensional printing is employed as a transformative tool for biomedical applications. It can realize the aim of culturing cells in 3D systems by mimicking the spatial features of native ECM, providing a more physiologically appropriate environment in which to regulate cell behaviors [61]. However, to date, it has been impossible to fully achieve the distinct interwoven network of fibers with varied diameters. No approach can reproduce the biological molecules in the ECM [59]. Even though the critical roles played by the structure, mechanics, and topology of the ECM in regulating cell behavior are demonstrated, different types of tissue or organs have distinct spectra of biological components. It is still a challenge to produce synthetic mimics of NECMs.

NECMs can induce constructive remodeling, and their wide clinical applications are a result of their bio-inductive properties [62]. The prerequisites of rapid host cell infiltration and proliferation can be met in ECM scaffolds [63]. Some ECM scaffolds not only provide for host cell attachment and the migration of keratinocytes, fibroblasts, and other cells implicated in wound healing, but facilitate the rapid infiltration of functional host cells. Moreover, marrow-derived stem cells may be recruited to the wound site in response to the ECM scaffold, differentiating into various cell types that generate original host tissue instead of scars [64,65]. Although the question of how ECM scaffolds facilitate the constructive remodeling of tissues is still to be elucidated, the innate bio-inductive properties of ECM scaffolds play a critical role in tissue remodeling and regenerative medicine applications. Some of these NECMs have shown great promise in the realm of regeneration medicine, such as in wound repair and tissue engineering. Due to their distinguished biocompatibility, bioactivity, and mimicking of the biochemical and biophysical cues of the native tissue properties, they are widely exploited and applied in many different repair and regeneration scenarios for damaged organs or tissues.

## 4. Origins, Degradability, and Physical Properties

### 4.1. Origins

**Figure 2:**
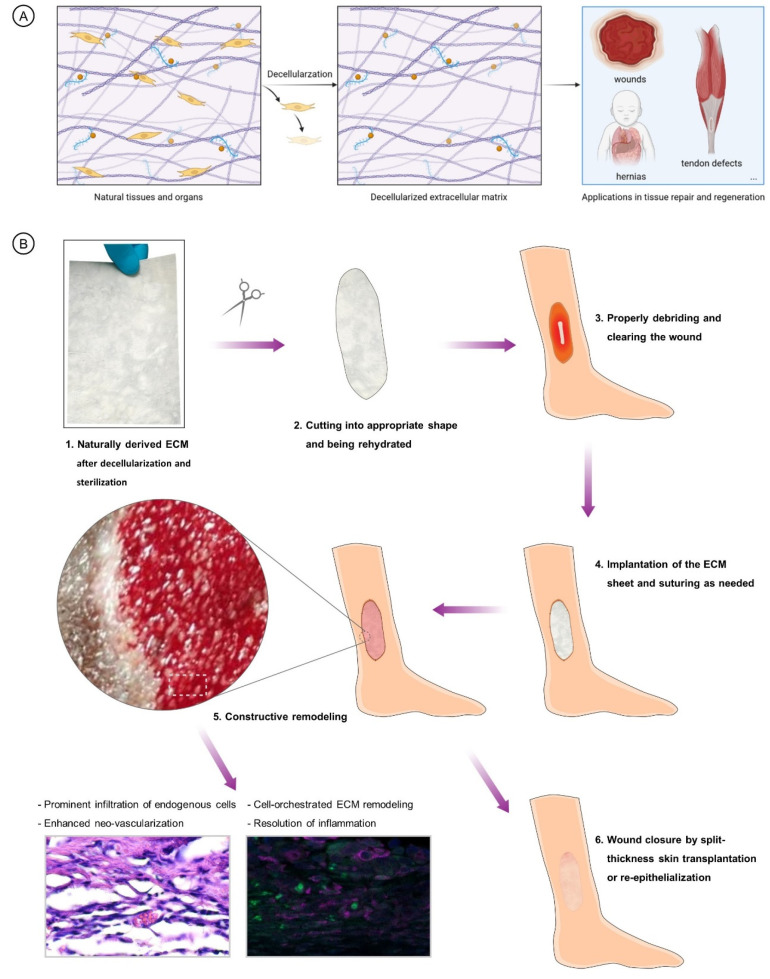
(**A**). Brief illustration of the preparation and application of NECM materials. (**B**). Classic procedures of NECM application in treating limb ulceration and the ensuing mechanisms of constructed remodeling and repair of the wounds.

The animal species sources, source tissue, names, and abbreviations are shown in Table 1. NECMs have been derived from animal tissues, such porcine or bovine dermis or urinary bladder. Generally, the major procedures of deriving ECMs from different types of tissues include tissue isolation, decellularization, drying, sterilization, and post-processing (Figure 2A). Decellularization can be achieved using chemical, physical, and/or enzymatic approaches. Recently, ECMs have been isolated from human sources such as placental tissues [66]. The NECM is widely utilized in different realms of medical use, including tissue engineering, tissue or organ reconstruction, the reinforcement of tissue defects, drug delivery, and immunological research. In in vivo tissue engineering, NECMs are used as matrices guiding tissue reconstruction and regeneration or implants replacing native tissues’ physical function [67]. As exemplified by the application of NECM in lower limb ulcerous wounds, the implantation of the material initiates the process of constructive remodeling which features in the prominent infiltration of endogenous cells, neo-vascularization, cell-orchestrated remodeling, and the resolution of inflammation (Figure 2B).

### 4.2. Degradability

**Figure 3:**
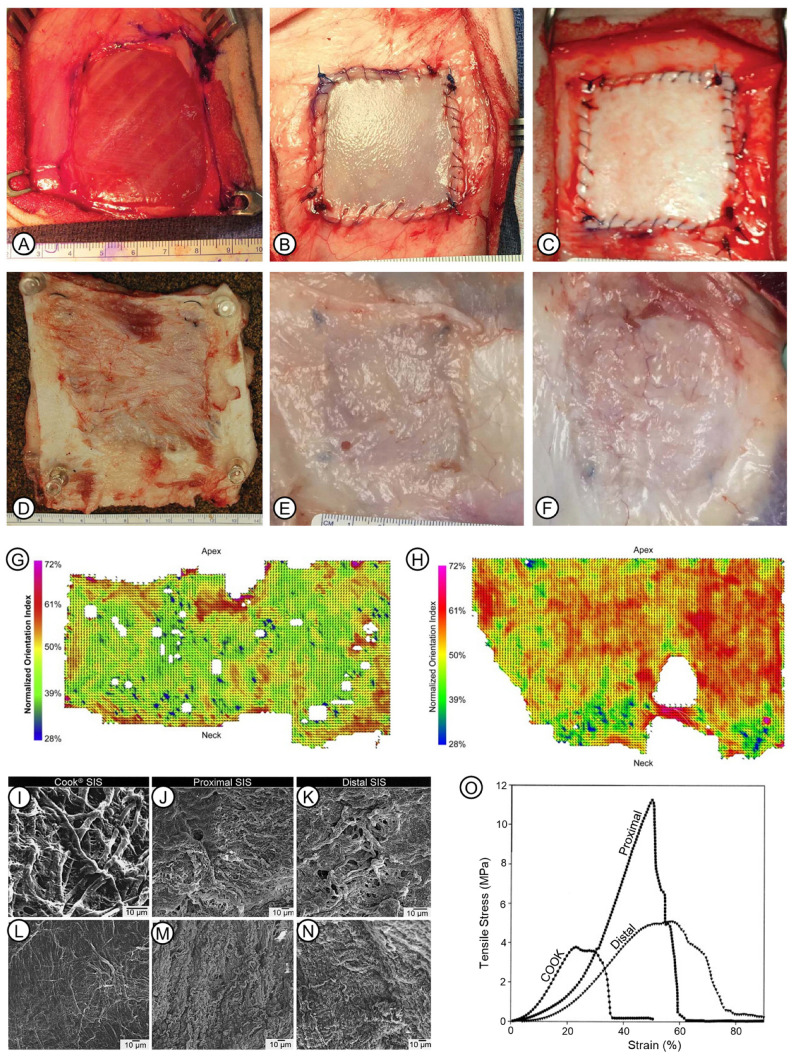
Some features of the biodegradability and physical properties of naturally derived ECM. (**A**–**F**): Appearance of ADM and UBM with surrounding tissue after being implanted in a fascia lata defect of the sheep model. Reprinted with permission from Ref. [72] under the terms of the Creative Commons Attribution License 2018, Young et al. The defect was deep in the vastus lateralis muscle after the excision of the fascia lata (**A**). Immediately, UBM (**B**) or ADM (**C**) was implanted into the defect. At 3 months after surgery, control defects showed the generation of fibrotic tissue (**D**); UBM showed complete remodeling and integration with the surrounding tissue (**E**); and the outline of ADM could still be observed (**F**). (**G**,**H**): Reprinted with permission from Ref. [89] 2008, Gilbert et al. Typical plot of collagen fiber orientation of UBM after being scraped circumferentially (**G**) or longitudinally (**H**). (**I**–**N**): Microscopic morphology of various SIS scaffolds. The serosal surface of Cook^®^ SIS was shown (**I**–**K**). Microscopic morphology of mucosal-side surface of Cook^®^ (**D**); proximal (**M**), and distal (**N**) SIS are shown. (**O**): Stress–strain curves of Cook^®^, proximal, and distal SIS. (**I**–**O**): Reprinted with permission from Ref. [90] 2005, Raghavan et al.

The NECM is biodegradable. It can be remodeled and degraded by host cells and enzymes secreted by these cells [68]. However, the degradation behavior is determined by the type of NECM, the specific site of NECM implantation, and the individualized medical condition of the patient. In the remodeling process, NECM degradation can provide space for neo-tissue formation. Tissue sources may play a key role in determining the degradation rate of NECM grafts. For instance, in the scenario of full-thickness burn wounds, bADM was not degraded but rapidly assimilated by the patient’s cells and growth factors to facilitate angiogenesis and dermal regeneration [69,70], which is in contrast to UBM and SIS, which are processed into multi-layer forms to persist in the wounds and match the time of wound healing. Generally, ADM in its non-crosslinked form is degraded more slowly than SIS and UBM [71]. As tested in the sheep model of the fascia lata defect, the morphology and color of UBM was changed with a diminished outline three months after implantation (Figure 3E), during which the morphology and color was retained for ADM (Figure 3F) [72]. Animal age is another variable that influences NECM degradability. Older SIS showed higher elastic moduli, tensile strength, and thickness, which rendered its degradation slower [73]. SIS grafts derived from 3- to 52-week-old pigs were tested using a rat abdominal wall reconstruction model [74]. SIS grafts derived from young pigs showed an enhanced anti-inflammatory response, and this effect decreased with age increase [75]. Therefore, the biodegradability of NECMs is an important factor to consider both when using the materials for medical applications and in designing new-type ECM devices.

The degradation of NECM grafts is started by neutrophils [75]. Neutrophils secret metalloprotease (MMP)-8 and -9 to prepare the grafts for the binding of monocytes and T cells [76]. When macrophages arrive, they secret MMP-1, -3, -7, -9, and -12 to further degrade the grafts [77]. During matrix degradation, cytokines are released upon degradation or confirmation change in ECM molecules [78]. However, these released cytokines need further activation or degradation by MMPs [78]. Moreover, macrophages themselves secret transforming growth factor-β1 and tissue inhibitors of metalloprotease to inhibit or control MMP activity [79].

Degradation is of much relevance to in situ tissue regeneration. A proper degradation rate is required to provide space for neo-tissue formation, which in turn helps to resolve inflammatory responses [80]. In a rat model of abdominal wall defects, SIS and UBM outperformed all the other tested NECM grafts, including pADM, regarding reconstructive remodeling [71]. This is likely the consequence of slower rates of pADM as compared with UBM and SIS [72,81]. The early infiltration of immune cells including macrophages and T cells and increased degradation are correlated with a better performance of NECM grafts [82]. Immune-cell-mediated degradation led to the release of chemoattractants, growth factors, and extracellular vesicles preserved in the NECM, which recruit local and circulating progenitor cells and enhance neo-tissue generation [83,84,85].

Cryptic peptides produced by NECM degradation, named “matrikines” within native ECM, regulate the behaviors of immune cells. Macrophages exposed to degradation products of various porcine tissue-derived ECM scaffolds showed a unique phenotype that was associated with the suppression of inflammation and high antigen-presenting capabilities [86], even in a harsh pro-inflammatory microenvironment such as in volumetric muscle loss [87]. Degradation products of SIS directly activated constructive macrophage polarization, which was similar to the macrophage polarization induced by IL-4 [88]. ECM degradation products have also been shown to initiate cross-talk between macrophages and T regulatory cells, a critical determinant of downstream remodeling outcomes [86].

### 4.3. Physical Properties

Physical properties of naturally derived ECM, such as topology, ultrastructure, mechanical strength, and the three-dimensional arrangement pattern of collagen fibers, are key parameters that have a fundamental effect on the performance of the scaffolds. Their features vary depending on the source tissue and extraction and processing procedures. Topology refers to fiber alignment, orientation, and aggregation in the ECM. It is mostly determined by the origin type of the ECM, and also influenced by anatomical locations and processing methods. UBM scraped along the circumferential directional showed a more homogenous orientation of fibers, as compared with the direction along the bladder longitudinal axis (Figure 3G,H) [89]. The serosal side of Cook^®^ SIS was more fibrous than samples from the proximal and distal sides of the small intestine (Figure 3I–N) [90]. The dense and compact surface topology is correlated with the arrangement of robust collagen fibers in the dermis. Conversely, the UBM and SIS have interlaced, fine collagen fibers that may be more effective for cell attachment and cell triggered biodegradation, which further produces chemotactic molecules. It is also suggested that the surface topology is able to regulate cell plasticity in conjunction with other coordinating properties, such as the surface ligand and the stiffness of the matrix [91].

The thickness and mechanical strength of uni-layer NECMs are shown in Table 2. Both SIS and UBM have significantly lower tensile strength than ADMs, whereas they all have comparable elastic modulus except for SIS [92,93]. In the same sample, mechanical strength may be varied due to the locations of the materials. The tensile strain at break of the distal samples was higher, as compared with proximal samples and Cook^®^ SIS samples (Figure 3O) [90]. Although it remains to be investigated whether the mechanical strength is correlated with the performance of ECM in tissue repair, the high relevance between ECM mechanical strength and its degradation behavior has been confirmed.

## 5. Immune Response to NECMs

The immune response of NECMs as allo- or xenografts is of great relevance to graft performances in wound or injury sites due to the possibility of graft repulsion caused by foreign body reaction or an unsatisfactory tissue-reconstruction effect as a result of the graft’s lack of appropriate immunomodulatory function. The aim of decellularization is to remove the cellular components as much as possible to prohibit the occurrence of a foreign body reaction, while at same time retaining the native structure and matrix-bound cytokines and growth factors [75]. However, the immune response to NECMs is difficult to define due to the complexity of NECM material variables, including animal species, tissue sources, and the age of the tissue source, and also the permutations of these variables [81]. Certain NECMs such as UBM and SIS exhibit distinct immune responses characterized by site-specific constructive remodeling, rather than the classical pattern of foreign body reaction [71]. In addition, the contribution of T cells in NECM graft-induced tissue repair and reconstruction was confirmed in the murine model of corneal wound healing [94] and the rate model of abdominal wall defects [71]. The understanding of both innate and adaptive systems in the NECM response and their contribution to in situ tissue regeneration has been evolving rapidly, which guides the development of new grafts.

### 5.1. Innate Immune Response

Due to the complexity of various kinds of components in NECMs, protein-to-protein interactions between serum and an NECM are difficult to fully examine [95]. As with artificial materials, certain serum proteins such as von Willebrand Factor (vWF) and soluble fibronectin adhere to the grafted NECM, forming a provisional matrix [96]. However, a prominent feature for NECMs is they have specific sites for cellular adhesion [97]. After the provisional matrix forms, neutrophils arrive and act to clear any pathogens. They may also impact the subsequent immune responses by releasing cytokines. Monocytes start to enter the peri-graft zone 48 h after grafting. There, differentiated monocytes, macrophages, play versatile roles in determining the outcome of graft–tissue interaction [98]. Macrophages display a reparative phenotype in the process of constructive remodeling that facilitates in situ tissue regeneration [87,99]. Macrophages also play key roles in the degradation of NECM grafts [100].

The polarization of macrophages toward anti-inflammatory states is considered to be correlated with positive outcomes of NECMs-facilitated tissue repair, characterized by constructive remodeling [101]. Among fourteen commercialized NECM grafts tested in a rat model of abdominal wall defects, SIS and UBM, as compared with pADM, displayed significantly better histologic scores than were correlated with their earlier M2 polarization [71].

Damage-associated molecular patterns may be present in NECM grafts, including residual DNA, high mobility box group 1, and heat shock proteins [102,103]. Among them, residual DNA, detected in a number of NECMs, is recognized as damage-associated molecular pattern by macrophages [104,105]. It is hypothesized that a threshold level of residual DNA in NECMs exists, and the use of NECMs is considered to be safe under this level [80,106]. However, this threshold level is not ideal or standardized, and further study is needed to demonstrate whether such a threshold level exists regarding the patterns of the innate immune responses invoked by NECMs.

It is possible that not all the DAMPs present in NECM grafts invoke negative responses. High mobility box group 1 may promote constructive remodeling by inducing anti-inflammatory responses [103]. In addition, many NECM molecules such as fibronectin, heparan sulfate, and hyaluronic acid have been suggested to act as DAMPs, especially when fragmented [102]. Moreover, it is to be confirmed whether residual RNA acts as a DAMP [107]. Further studies are needed to understand the role of different molecules in the immune responses to NECM grafts.

### 5.2. Adaptive Immune Response

As compared with natural or synthetic polymer-based grafts, NECM grafts have more possible antigens due to the complexity of their components. Both allogeneic and xenogeneic NECM grafts activate adaptive immune responses. As for humoral immunity, allogeneic grafts generally show negative responses [108]. Due to the animal origin, xenogeneic grafts may activate humoral responses. Adaptive immune responses may have positive effects on NECM-graft-mediated constructive remodeling. Several studies have shown the beneficial effect of T helper cells modulated by NECM grafts [94,109,110]. In murine cornea wounds, UBM promotes type 2 immune responses characterized by the upregulation of IL-4 expression in peripheral CD4^+^ T cells [94]. Subcutaneously implanted SIS and UBM increased the local T cell-dependent IL-4 expression, which was a main reason for the lack of graft rejection [109]. Also, it was found that this graft-mediated immunomodulative effect was not systemic, rendering SIS and UBM applicable to immunocompromised patients [110].

Two types of humoral responses to NECM grafts were investigated; anti-Gal antibodies and anti-non-Gal antibodies are produced in response to α-Gal and other non-α-Gal epitopes. Sugar moieties that are present in the microbiome of human bodies and all non-primate mammals induce the production of anti-Gal antibodies [111]. When grafted to man-made abdominal defects of African Green monkeys, wild-type SIS containing the Gal epitope increased the level of serum anti-Gal antibodies, as compared with SIS derived from GalKO pigs [112]. However, no negative effect on tissue remodeling was observed for GalKO SIS [110]. The production of anti-non-Gal antibodies is not related to α-Gal. Various decellularization methods produce residual MHC, potent non-α-Gal antigens, in NECM grafts [113]. It was postulated that an excessive level of serum antibodies against NECM antigens may hinder the migration of stem cells into NECM grafts due to the decrease in adhesion sites [111].

## 6. Clinical Applications

### 6.1. UBM

#### 6.1.1. Wound Healing and Skin Repair

To treat both chronic and acute wounds, UBM is transplanted to a clean wound after appropriate debridement. In comparison to Dermagraft, a tissue-engineered product containing living fibroblasts, UBM provided similar results for the healing of diabetic foot ulcer wounds, but with reduced economic expenses, as indicated by an interim analysis in a randomized controlled trial [114]. UBM coverage has been shown to be efficacious for the treatment of small complex wounds of orthopedic trauma patients. It can be performed without delay, a key drawback faced in flap grafting [115]. The easy-to-handle property of UBM rendered it fit for wounds with exposed tendons or bones, distal leg wounds, and open foot fractures, which require complex reconstruction surgery or extended negative pressure wound therapy [116].

**Figure 4:**
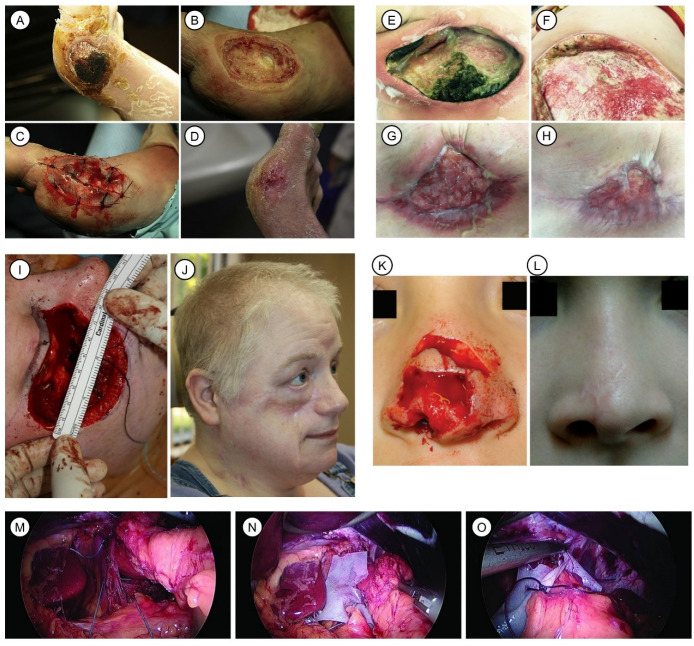
Clinical applications of UBM in tissue repair and reconstruction. (**A**–**D**): UBM treatment of a diabetic foot ulcer wound. Reprinted with permission from Ref. [117] 2010, Kimmel et al. Necrotic tissue is visible in the medial of the right foot of the patient (**A**). Debridement was operated deeply to the joint capsule (**B**). Hydrated, fenestrated UBM sheet implanted with suturing to the wound edge (**C**). On 13 weeks after the implantation, the wound was closed (**D**). (**E**–**H**): Repair of a *Pseudomonas aeruginosa*-infected wound resulting from right breast mastectomy by UBM. Reprinted with permission from Ref. [121] under the terms of the Creative Commons Attribution License 2017, Puckett et al. The wound is heavily infected by refractory *Pseudomonas aeruginosa* (**E**). Three days after the implantation of UBM, with the micronized powder form and sheet simultaneously, the infection decreased and granulation tissue formed (**F**). About two months after weekly UBM treatment, the wound area reduced significantly (**G**). Approximately three months after UBM treatment, the wound contracted and was near to full closure (**H**). (**I**,**J**): Reconstruction of maxillectomy defect. Reprinted with permission from Ref. [125] under the terms of the Creative Commons Attribution License 2013, Kruper et al. The facial defect was achieved by resection of squamous cell carcinoma (**I**). The wound was repaired with UBM, and the smooth texture of the reconstructed skin was visible. (**K**,**L**): Reconstruction of pediatric nasal defect due to dog bite avulsion. Reprinted with permission from Ref. [126] 2020, Ong et al. The nasal tip, right ala, and dorsum were injured (**K**). Good appearance of the reconstructed nose was evident after 4 months of UBM treatment and pulsed dye laser therapy (**L**). (**M**–**O**): Use of UBM in the treatment of large hiatal hernia. Reprinted with permission from Ref. [127] under the terms of the Creative Commons Attribution License 2019, Sasse et al.

To date, there is a clear scientific rationale for using NECMs in calcitrant chronic wounds, including pressure ulcers, diabetic foot ulcers, venous leg ulcers, radiation ulcers and even heavily infected wounds. Clinical evidence supporting the use of NECMs has grown over the past several decades. Severe, chronic wounds that did not heal well after receiving basic wound care and that were repeatedly treated with UBM showed complete epithelialization. UBM showed a promising solution to the therapies of recalcitrant chronic ulcers (Figure 4A–D) [117]. UBM allows for the healing of complicated open wounds that do not respond well to conventional therapy [118]. The treatment of wounds with exposed tendons results in a more stable and less scarred wound that more closely resembles normal foot and ankle appearance, as compared to prolonged negative pressure wound therapy [119]. A retrospective review that evaluated 34 patients suggests that UBM is effective in the treatment of acute and recalcitrant diabetic or venous ulcerations [120]. The successful healing of a Pseudomonas infected wound was achieved by using UBM directly for a 52-year-old female with an obesity problem (Figure 4E–H) [121].

Treatment with UBM in its micronized powder form enhanced the healing and promoted the epithelialization of chronic wounds caused by radiation, which failed to heal using the traditional method of wound treatment [122]. This clinical finding is further supported by results obtained in a controlled test that evaluated the effect of topical UBM on irradiated wounds in a murine model [123]. UBM micronized powder and sheets used together allowed wound healing over bones exposed after distal amputation executed to treat diabetic foot infection, which successfully prevented the proximal amputation of two diabetic foot patients [124]. UBM grafting initiated epithelialization and promoted the healing of nonhealing radiation wounds [122].

#### 6.1.2. Plastic and Surgical Reconstruction

Cases receiving UBM grafting to reconstruct special tissues were reported. The salvaging of the failed regional flap was accomplished by using UBM in a case of maxillectomy defect due to the resection of squamous cell carcinoma (Figure 4I,J) [125]. Avoiding donor site morbidity, UBM provided a safe and effective solution to the head and neck reconstruction of injury in pediatric patients by dog bite avulsion (Figure 4K,L) [126].

#### 6.1.3. Hernia

Diaphragmatic reinforcement with UBM allowed the repair of a laparoscopic hiatal hernia with reduced recurrence [127]. UBM reinforcement facilitated the repair of a large hiatal hernia concomitant with a sleeve gastrectomy surgery (Figure 4M–O) [128]. UBM-reinforced repair of paraesophageal hernia was associated with fewer recurrences of severe symptoms as compared with primary repair [129].

### 6.2. Porcine Small Intestinal Submucosa (SIS)

#### 6.2.1. Wound Healing and Skin Repair

**Figure 5:**
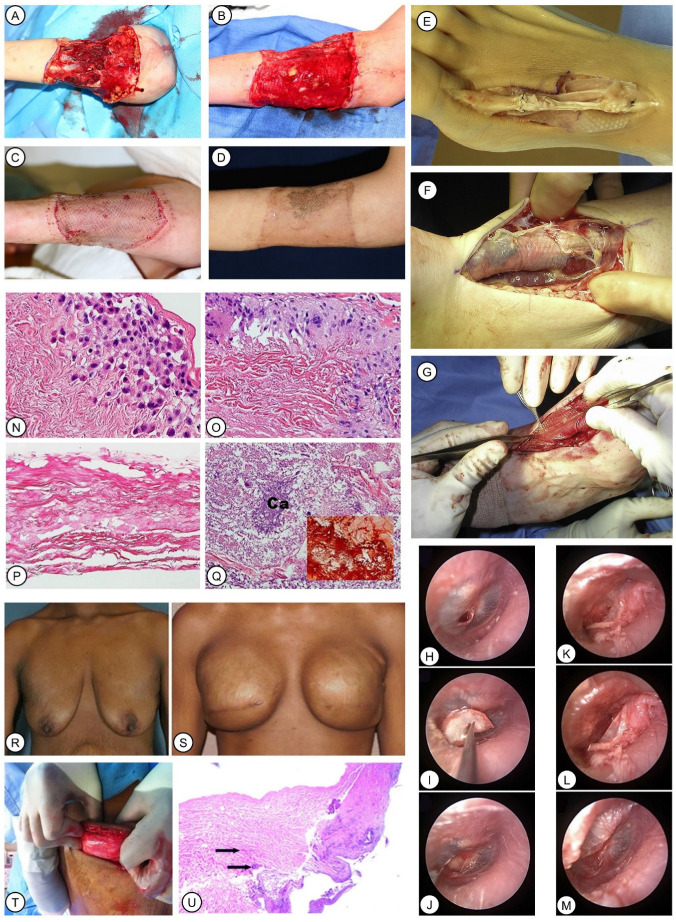
Classic examples of the application of SIS and ADM. (**A**–**D**): Treatment of a dog bite wound on the patient’s forearm. Reprinted with permission from Ref. [132] 2019, Collini et al. Extensive debridement was performed (**A**). Granulation tissue was visible 12 days after SIS implantation (**B**). Skin autograft was transplanted (**C**). Good skin texture and color were observed 3 months after initial injury (**D**). (**E**–**G**): SIS was utilized in foot and ankle reconstruction to prevent the adhesion of the tendon with paramount effect. Reprinted with permission from Ref. [136]. H-M: Repair of pediatric tympanic membrane perforation using SIS. Reprinted with permission from Ref. [137] 2017, Basonbul et al. SIS was inserted with a method of underlay myringoplasty (**H**–**J**). The flap was conveniently folded back over the graft using SIS positioning during underlay tympanoplasty (**K**–**M**). (**N**–**Q**): Histological examination of an SIS patch used in pediatric congenital heart surgery. Reprinted with permission from Ref. [143] 2016, Woo et al. Infiltrated plasma cells and lymphocytes in SIS were observed **(N**). Foreign-body giant cell reaction was visible in the interface between SIS and the surrounding cardiac tissue (**O**). Degeneration of SIS collagen was observed in some samples, as shown (**P**). Calcification occurred in intensive degenerated fibers in some cases (3/12 cases) (**Q**). (**R**–**U**): Use of ADM in breast reconstruction. Reprinted with permission from Ref. [150] under the terms of the Creative Commons Attribution License 2014, Bullocks et al. The patient had immediate breast reconstruction after bilateral mastectomy (**R**). Better appearance was observed 7 months after a second reconstruction with concomitant implantation of tissue expanders and ADM (**S**). Integration of the ADM into the surrounding tissue was visible (**T**). Histological examination of biopsy of one patient showed cell ingrowth (arrows) into the ADM matrix (**U**).

SIS has been demonstrated, by primary studies, to aid in the growth of dermis-like tissue in both complex acute and recalcitrant chronic wounds. It is thought to be capable of accelerating wound closure, avoiding donor-site complications, and enhancing granulation in locations of limited blood supply, thus improving graft-take, reducing dehiscence, and supporting the uptake of skin flaps [130]. In addition to acting as a scaffold for dermal repair, SIS stimulates changes in the wound micro-environment, resulting in more prominent epithelial maturation [131]. It was utilized with the bolster technique, in which moistened cotton was sutured to create the bolster dressing in order to maintain SIS adherence to the wound bed, which assists with the integration of SIS into complex wounds, including a dog-bite forearm wound with intensively exposed muscles and tendons (Figure 5A) [132]. Notably, granulation tissue grew fast in the wound, and 100% graft-take was achieved (Figure 5B–D) [132].

#### 6.2.2. Hernia

SIS helps to reduce the recurrence rate when applied as a prosthesis to assist in the repair of laparoscopic paraesophageal hernia, as confirmed in a randomized controlled trial [133]. Via laparoscopy, SIS grafting can be implemented to treat ordinary hernias, whether with contamination or not, leading to a reduced recurrence rate [134]. However, it is also suggested that repair using SIS gives rise to a high recurrence rate after resection or excision surgery [135]. As compared with its artificial counterparts, SIS significantly reduced the median time to full recovery of hernias, even though complications including pain and discomfort were also more obvious [95].

#### 6.2.3. Defects in Other Tissues and Organs

Regarding the performance of SIS in foot and ankle reconstruction, an excellence rate of 85.19% (46 in 54 cases) was observed in a retrospective cohort study [136]. SIS effectively prevented peritendinous adhesions and rendered the reconstructed or allograft tendons capable of mobilizing and gliding (Figure 5E–G) [136].

An SIS graft was used in both endoscopic myringoplasty (Figure 5H–J) and underlay tympanoplasty (Figure 5K–M) [137]. Precise and delicate notches can be easily made in SIS, which resulted in greater convenience and reduced the difficulty of surgical operations.

In treating congenital epispadias in children, along with severe penile curvature, SIS grafting on the corporal body facilitates epispadias repair with satisfactory cosmetic effect and a relatively normal penis appearance [138]. SIS grafting may help to lower the incidence of recurrent chordee, which is a frustrating complication following ventral lengthening for the treatment of proximal hypospadias [139]. SIS was also used to treat severe penis curvature with a low risk of complications, as suggested [140].

Used in cardiovascular surgery, SIS is safe for the repair of ventricular and atrial defects [141]. Following cardiac surgery, pericardial closure using SIS is correlated with a decreased incidence rate of such complications as pleural effusion and pericardial effusion, as compared with data nationwide [142]. Even though histologic evidence suggested that SIS is not capable of integrating into cardiac wounds (Figure 5N–Q), it is demonstrated to have a favorable effect on pediatric patients having cardiac surgery [143].

SIS facilitates the rapid closure of renal defects caused by tumor nephrectomy [144]. It can further inhibit bleeding after surgery and show much promise in encouraging patients with tumors more than 4 cm in size to accept the surgery [144].

Despite higher expenses, SIS decreased the time to healing in patients who underwent cervicovaginal reconstruction, as compared with a split-thickness skin graft [145]. SIS can be completely remodeled and replaced by newly generated tissues, including skeletal muscle [146].

### 6.3. ADM Derived from Human Cadaver Skin, Fetal Bovine Dermis, and Porcine Dermis

#### 6.3.1. Breast Reconstruction

Breast reconstruction surgeries based on tissue expander implants account for nearly 60% of all post-mastectomy breast reconstruction [147]. The use of ADMs allows an appropriate subcutaneous fold to be established, thus preventing the lifting of the rectus and serratus fascia to form pockets for implants. It is clinically verified that the use of ADMs reduces the magnitude of foreign body reaction, contracture, and surface roughness of the implants [148]. hADM induces a lower lever of inflammation and granulation formation than native implants, indicating great potential for the prevention of capsular contracture, a common complication of implant-based breast reconstruction [149]. For example, at 5 months after permanent breast implantation, hADM with 97% DNA removed integrated with the surrounding tissue, and granulation took place on its surface (Figure 5R–T), which was further confirmed by a histological examination of the specimen from another case (Figure 5U) [150]. This facilitates the coverage of the implants without autologous soft tissue, of which there is often a shortage in such a circumstance.

The use of pADM and bADM in breast reconstruction and deformity correction is compared with hADM in a controlled clinical trial [151]. A comparative study suggested that bADM and hADM showed similar rates of major complications after breast reconstruction [152]. Although further research on the safety and efficacy of bADM is needed, bADM has great potential for lowering costs. Similar results were obtained for pADM on its use in immediate breast reconstruction, including comparable rate of complications [151].

#### 6.3.2. Wounds

hADM can significantly reduce the area of venous-leg-ulcer wounds [153]. hADM-treated diabetic foot ulcers had a 69.6% rate of complete closure as compared to 46.2% with advanced moist wound therapy [154]. The results of a randomized controlled trial suggested that when used concomitantly with standard of care, bADM enhance the healing of diabetic foot ulcers [155]. In a cohort study, bADM achieved 76% diabetic-foot-ulcer wound closure at about 8 weeks [156]. In addition, bADM, such as PriMatrix^®^ dermal repair scaffold derived from fetal bovine dermis, is widely used to treat severe burn wounds.

#### 6.3.3. Reconstructive Gynecology

hADM was used in paravaginal vaginal reconstruction to correct dislocations of primary or recurrent vaginal dislocations [157]. Follow-up data of 24 of 33 patients showed that the reconstruction with hADM was safe and well-tolerated [157]. Similar results were obtained in a study on hADM-treated vaginal wall prolapse [158]. In laparoscopic sacrocolpopexy, hADM is also shown to be more effective than polypropylene mesh, with no erosion complications and significant patient satisfaction regarding their prolapse treatment and current health status.

#### 6.3.4. Hernia

ADMs are used to close major abdominal wall incisions post abdominal surgery or after incisional hernias occur. As compared with non-absorbable meshes, ADMs are more feasible for providing sufficient tensile strength to prevent hernias. Moreover, ADMs can be used in infected fields where the incidence rate of hernias is high [159].

#### 6.3.5. Other Defects

pADM was used to augment the repair of full-thickness rotator cuff tears traditionally treated by arthroplasty and tendon transfers [160]. It was also used to cover the exposed bone in irreparable rotator cuff tears to relieve pain. The injection of micronized acellular dermal graft helped rejuvenate the aged lips [161].

ADMs were also used to augment penile dysfunction and treat erectile dysfunction, with decreased rates of necrosis, reduced surgery times, and improved surgical subtlety [162].

## 7. Indications, Features, and Clinical Relevance of Some Marketed NECM Devices

The names, sources, and appearances of some marketed NECM devices are summarized in Table 3, with their indications, features, and clinical relevance briefly introduced.

## 8. Conclusions

Bioactive materials based on NECMs show significant capabilities to promote in situ tissue regeneration in the merits of their inherent nano-structures, biosignalling cues, and degradability. Nano-structures of NECMs enhance cellular functions such as cell attachment, proliferation, and differentiation. They also mediate the bidirectional transmission of mechanical information between cells and scaffolds. Better performance of NECM grafts in constructive remodeling is related to an earlier infiltration of the immune cells, including macrophages and T cells and scaffold degradation. SIS and UBM induce anti-inflammatory macrophage M2 polarization and the expression of IL-4 by T cells. These properties render NECM biocompatible and potent for promoting in situ tissue regeneration, which is supported by clinical evidence.

## Figures and Tables

**Table 1:** Names, abbreviations, animal species sources, and source tissue of NECM grafts.

Name of NECM Graft	Abbreviation	Animal Species Source	Source Tissue
urinary bladder matrix	UBM	porcine	the base membrane and lamina propria of urinary bladders
small intestinal submucosa	SIS	porcine	the submucosa of the small intestine
fetal bovine acellular dermal matrix	bADM	fetal bovine	dermis
porcine acellular dermal matrix	pADM	porcine	dermis
human acellular dermal matrix	hADM	human	dermis
acellular human amniotic membrane	AHAM	human	amniotic membrane
bovine pericardium extracellular matrix	BP ECM	Bovine	pricardium

**Table 2:** Thickness and mechanical strength of uni-layer NECMs.

Type of NECM	Uni-Layer Thickness (mm)	Normalized Force at Rupture [10 N/mm^2^]	Strain
SIS [90]	0.25 ^&^	3.76 ^&^	0.23 ^&^
UBM [92]	0.15	0.29	0.40
hADM [93]	1.11	17.7	0.45 ^#^
bADM [93]	1.76	23.8	0.48 ^#^
pADM [93]	1.57	21.0	0.31 ^#^

^&^: hydrated; ^#^: at 10 N/mm^2^ stress.

**Table 3:** Information on some marketed NECM devices.

Name	Source	Appearance	Trade or Marketed Names	Indications	Feature and Clinical Relevance
Urinary bladder matrix	Porcine bladder	Membrane	Cytal^®^ wound/burn matrix	- Complex, acute, and/or surgical wounds - Traumatic wounds - Chronic wounds - Surgical or plastic reconstruction - Hernia repair - Peripheral nerve repair, etc.	- Supporting soft tissue repair for both acute and chronic complex wounds and burns - Advanced methods for plastic and reconstructive surgery and complex hernias - Enhancing regeneration of nerves and tendons
Urinary bladder matrix	Porcine bladder	Particle	MicroMatrix^®^ micronized particles	- Acute wounds - Different kinds of ulcers - Wound dehiscence - Trauma wounds	- Complete context with wound beds
Acellular dermal matrix	Fetal bovine dermis	Membrane	PriMatrix^®^ dermal repair scaffold	- Acute wounds - Different kinds of ulcers - Wound dehiscence - Trauma wounds	- Rich in Type III collagen
Small intestinal submucosa	Porcine jejunum	Membrane	OASIS^®^ Matrix Products; Biodesign^®^	- Acute wounds - Different kinds of ulcers - Wound dehiscence - Trauma wounds	
Acellular dermal matrix	Human cadaver skin	Membrane	AlloDerm^TM^ RTM	- Repair or replacement of damaged or inadequate integumental tissue or for other homologous uses of human integument	- Most used ADM - Positive recognition - Supporting regeneration - Revascularization - Fibroblast repopulation and reduced inflammatory response
Acellular dermal matrix	Human cadaver skin	Particle	Cymetra	- Lip augmentation - Nasolabial folds - Corner of the mouth - Dermal defects - Facial lipoatrophy	- In micronized or injectable form
Amniotic allograft membrane	Human amniotic tissue	Membrane	AminoExcel^®^	- Wound covering	- Excellent handing and non side specific application
Acellular dermal scaffold	Porcine dermis	Membrane	STRATTICE™ Reconstructive Tissue Matrix	- Soft tissue damage or rupture - Inguinal hernia repair - Parastomal hernia repair - Abdominal wall repair	- Reinforces soft tissue where weakness exists - Surgical repair of damaged or ruptured soft tissue membranes
Acellular dermal scaffold	Porcine dermis	Membrane	CollaMend^®^	- Repair of hernia - Reinforcement of plastic and reconstructive surgery - Repair of hernia and chest wall defects - Surgical repair of damaged or ruptured soft tissue membranes	- To reinforce soft tissue where weakness exists
Bovine pericardium	Bovine pericardium	Membrane	Collagen Solutions	- Heart valves - Vascular patches - Staple line reinforcement - Hernia repair and reinforcement - Dental membrane - Pelvic floor reconstruction	- Minimally processed or crosslinked - Multiple thicknesses and physical properties
Bioimplant for soft tissue repair	Horse pericardium	Membrane	OrthADAPT^TM^ bioimplant	- Rotator cuff injuries - Reconstruction of anterior shoulder	- Repair, reconstruct, augment, and reinforce soft tissue I tendons and ligaments - Does not cause any significant inflammatory response
